# Assessing the exertion required to induce breathlessness in a population with advanced cancer: matching measures to the level of physical function

**DOI:** 10.1186/s12904-018-0386-1

**Published:** 2019-01-10

**Authors:** Kahren M. White, Meera R. Agar, David C. Currow

**Affiliations:** 1 Occupational Therapist, Private Practice, Bexley North, NSW Australia; 2 IMPACCT, Faculty of Health, University of Technology Sydney, PO Box 123, Ultimo, NSW 2007 Australia; 3 National Cancer Symptom Trials (CST) Group, Faculty of Health, University of Technology Sydney, Ultimo, NSW Australia; 4 South West Sydney Local Health District, Liverpool, NSW Australia

**Keywords:** Exercise-induced breathlessness, Clinical research tools, Six minute walk test, Arm exercises, Cohort study

## Abstract

**Background:**

The aim of the study was to assess four evidence-based assessments utilising exercise challenges that induce breathlessness, each with progressively less demanding levels of exertion, which can be tailored to people with a range of functional capabilities in the setting of advanced cancer for research studies. Functional cut off points for these assessments have not previously been defined.

**Methods:**

A cross sectional study of four exercise tests attempted by all participants: 6 min walk test (6MWT); (derived) 2 min walk test (2MWT); arm exercises; and reading numbers aloud. Performance status (Australia-modified Karnofsky Performance Status (AKPS)), baseline breathlessness using the modified Medical Research Council (mMRC) breathlessness scale, and a visual analogue scale of intensity and unpleasantness of breathlessness were measured. Co-morbidity was codified using the Charlson Co-morbidity Index. Percentage of people completing each test by AKPS level of function and baseline mMRC breathlessness scores were quantified.

**Results:**

In the 68 participants, poorer function decreased the proportion of people able to complete the exercise tests. For completion rates ≥80%, of 6MWT and 2MWT, only people with an AKPS 70–90 had completion. For arm exercises, this included people with an AKPS as low as ≥50; and for reading numbers, it included people with an AKPS of 40 but not below.

**Conclusions:**

Walking tests have poor utility in people with high levels of functional impairment. For people with high levels of dependence, reading numbers should be used in evaluating exercise-induced breathlessness in research studies. These data also suggest that people’s exertional limitations have been under-estimated as cancer progresses.

## Background

Clinical research of breathlessness may include measuring the subjective symptom’s intensity and unpleasantness, as well as using objective measures of the exercise that the person can undertake before being stopped through breathlessness, fatigue or both. The performance during the test and time to stopping become objective measures relating to cardio-respiratory function. In advanced cancer, as physical function expectedly declines with progression of a person’s life-limiting illness, the exercise tests to induce breathlessness that can be practically used for research will differ from people with no functional impairment.

Functional capacity has been extensively explored in people with cardiopulmonary disease where reasonable functional status has been maintained. One of the core methods of objectively assessing how function is limited by breathlessness, fatigue or both has been with the 6-min walk test (6MWT) [1]. The 6-min walk distance (6MWD) has become the primary outcome measure used in pulmonary rehabilitation programs as well as chronic heart failure and pulmonary hypertension [1–4]. The 6MWD has been used in clinical research to quantify exercise capacity in people with a range of conditions, including cancer [5–7]. In populations that are frailer and unable to undertake a 6MWT, there remains limited use of objective exercise tests to quantify limitations due to breathlessness.

The aim of the study was to assess the use of four evidence-based exercise tests that induce breathlessness to define at which levels of function each of these tests would be best employed. This has not been previously systematically explored and quantified, with functional cut off points for these assessments never having been defined.

## Methods

This cross-sectional study was undertaken from November 2010 to October 2015. Given that this was an exploratory study, a convenience cohort of 68 people were recruited.

### Setting

Participants were recruited from Prince of Wales Hospital Departments of Oncology and Palliative Care, Randwick, New South Wales; Braeside Hospital Department of Palliative Care, Prairiewood, New South Wales; Southern Adelaide Palliative Services, Daw Park, South Australia; Calvary Health Care Palliative Care Services, Kogarah, New South Wales in Australia.

### Participants

Participants were eligible for inclusion in the study if they had a diagnosis of advanced cancer, defined as metastatic or advanced loco-regional disease, a predicted survival of greater than 7 days as assessed by the referring doctor, were over the age of 18, able to give informed consent and were English speaking.

Participants were excluded if they had an Australia-modified Karnofsky Performance Scale (AKPS) of less than 20, unstable angina, a documented myocardial infarction during the previous 1 month, resting heart rate of more than 120 beats per minute, systolic blood pressure > 180, diastolic blood pressure > 100 at the time of the test, an inability to complete bilateral arm exercises or significant communication problems. These exclusions were to ensure safety when completing the walk tests. The ability to complete bilateral arm exercises and communication issues were to ensure safety and baseline ability in completing the arm test and reading number aloud test.

### Protocol

The exercise tests were undertaken with participants once, taking a period of up to 90 min, as participants had 20 min of rest between each of the tests. Baseline breathlessness measures and performance status were recorded. Participants attempted each of the four exercise tests. If they were unable to attempt any of the tests due to functional limitation, this was recorded. Study procedures were standardised across all study sites through education for data collectors on how to complete each test by the primary investigator. A standard procedure booklet was also provided to each data collector to follow while undertaking each exercise test, assessment of breathlessness, performance status and comorbidity.

### Exercise tests undertaken

#### 6MWT and 2MWT

The 6MWT was originally developed as a reliable and reproducible assessment of sub-maximal exercise tolerance in respiratory disease [2]. Initially a 12-min walk test was used, however results were found to be valid and reproducible with a 6MWT, and a 2-min walk test (2MWT) was subsequently derived [5, 6]. This study measured 6MWD and 2MWD as the first two levels of exercise tests. The 6MWT and 2MWT were undertaken using the Pulmonary Rehabilitation Toolkit protocol [7].

#### Arm exercises

Wilcock et al [8] developed an upper limb exercise test to assess breathlessness in people with a cancer diagnosis. Their research demonstrated this arm exercise test was able to stimulate breathlessness for use as an outcome measure to assess the benefit of new treatments to manage chronic breathlessness for people living with cancer [8]. The measurement of this exercise test is with the participant moving an outstretched arm between two points 20 cm above and 20 cm below their shoulder height in time to an audible beat. The participant changes arms at one-minute intervals until they can no longer continue.

### Reading numbers aloud

Reading numbers aloud was developed as an exercise test to assess breathlessness in people living with cancer prior to and following medical interventions for their chronic breathlessness [9]. The measurement of this exercise test is in the number of numbers that can be read aloud, and the number of breaths taken in a one-minute period, with the procedure repeated up to 5 times. A higher number represents a longer time to breathlessness.

### Breathlessness measures

The modified Medical Research Council breathlessness scale (mMRC), an ordinal rating scale of breathlessness, and a visual analogue scale measuring intensity and unpleasantness of breathlessness were used to identify the level of breathlessness experienced by the participant in daily life prior to attempting the outcome measures. A higher score reflects greater breathlessness.

### Performance status

One of the most common clinician-assessed tools used to identify performance status in people living with advanced cancer is the Australia-modified Karnofsky Performance Status (AKPS) [10]. The AKPS correlates with prognosis in advanced cancer and reflects the level of physical support people need in their activities of daily living. AKPS was used in this research to identify performance status of participants at the time of completing the outcome measures, with a lower AKPS score representing poorer function.

### Co-morbidity

Co-morbidity was codified using the Charlson Co-morbidity Index, a valid and reliable tool that has shown relationships with mortality, disability and length of hospital stay [11, 12]. This score omitted cancer, as all participants had a cancer diagnosis.

### Statistical analysis

Descriptive statistics were used to describe the population. Percentage of people completing each test by level of function (AKPS) and by baseline breathlessness scores (mMRC) were quantified, with an arbitrary rate of 80% accepted as likely to be of utility in clinical research. Results for each test at each level were then defined, exploring the absolute values of people able to complete the test and the standard deviation of the measures found. No data were imputed.

## Results

Sixty eight people participated in the study (28 (42%) males); median 70 (range 37–93; Table 1). All people had cancer with primary sites largely reflecting the burden of advanced cancer (lung 19 (28%); prostate 9 (13%); colon/rectum 7 (10%); breast 6 (9%)). Baseline scores for breathlessness were measured using the modified Medical Research Council scale (mMRC 0 = 9; mMRC 1 = 13; mMRC 2 = 16; mMRC 3 = 10; mMRC 4 = 18; two missing; median 2.0; range 0, 4).

**Table 1:** Participants’ characteristics in a cohort study of people with advanced cancer

Domain	Measure	Mean	SD	Median	Range	n (%)
Age		69.9	12.7	70.0	37, 93	
Gender	Male					28 (41)
Body mass index (BMI)		25.1	5.2	24.2	17.0, 41.6	
Functional status	AKPS	55.0	14.7	60.0	20, 90	
Breathlessness	mMRC	2.2	1.4	2.0	0, 4	
	Intensity (NRS; 0–10)	2.7	2.4	2.0	0, 8	
	Unpleasantness (VAS; 0–100)	25.5	28.2	15.5	0, 100	
Co-morbidities	CCI	6.4	1.6	6.0	2, 11	

*SD* standard deviation, *AKPS* Australia-modified Karnofsky Performance Status, *ECOG* Eastern Co-operative Group performance status, *mMRC* modified Medical Research Council breathlessness scale, *NRS* Numerical Rating Scale, *VAS* Visual Analogue Scale, *CCI* Charlson Co-morbidity Index

Baseline performance status was measured using the AKPS (median 60; AKPS 90,80 = 7; AKPS 70 = 8; AKPS 60 = 22; AKPS 50 = 10; AKPS 40 = 17; AKPS 30,20 = 4). Functionally, the results equate to participants being able to care for most their activities of daily living, but requiring occasional assistance.

Co-morbidities related to cardiac or respiratory disease from Charlson co-morbidity index were relatively infrequent; COPD 6 participants; CCF and COPD 3 participants; CCF 2 participants; and one participant each with CCF and pulmonary hypertension, hypertension, angina, and asthma.

**Fig. 1:**
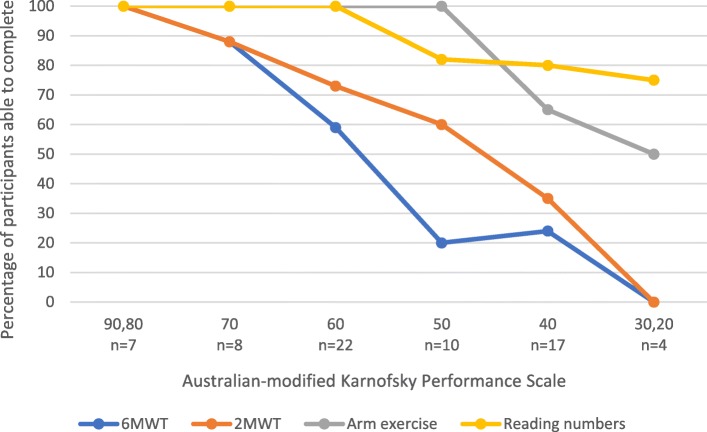
Percentage of completion of 6-min walk test, 2-min walk test, arm exercise and reading numbers by Australian-modified Karnofsky Performance Scale in 68 people with advanced cancer

As function decreased, the proportion of people able to attempt the exercise tests declined (Fig. 1). The authors have defined completion across all four exercise tests as those who attempted each test. A ceiling effect was noted for people with high function for the reading numbers and arm exercise tests. For completion of 6MWT and 2MWT, only people with an AKPS 70–90 had completion rates ≥80%. For arm exercises, this included people with an AKPS of ≥50; and for reading numbers, it included people with an AKPS of 40. These results define at which level of AKPS, that is performance status, each of the exercise tests is best utilised.

For values gained for people who completed each exercise test, the largest drops occurred between AKPS 60 and 50 for 6MWT and 2MWT, while for arm exercises there was no particular pattern of decline in values achieved by functional status and for reading numbers aloud. Similar results were seen from people with an AKPS 20–50.

The primary reasons for participants being unable to complete or participate in the exercise tests are listed in Table 2.

**Table 2:** Reason for participants’ being unable to complete exercise tests

Outcome measure	Reasons for not completing measure (more than 1 permitted)	n
6 min walk test	Reduced mobility	15
	Fatigue	9
	Breathlessness	7
	Participant declined	3
	Pain	2
	Lower limb oedema	1
2 min walk test	Reduced mobility	14
	Breathlessness	4
	Participant declined	3
	Fatigue	1
	Pain	1
	Lower limb oedema	1
Isometric arm	Pain	34
	General fatigue	22
	Arm fatigue	18
	Breathlessness	7
	Participant declined	2
Reading numbers	Fatigue	5
	Breathlessness	2

NB: Participants were able to give multiple reasons for being unable to exercise tests

**Fig. 2:**
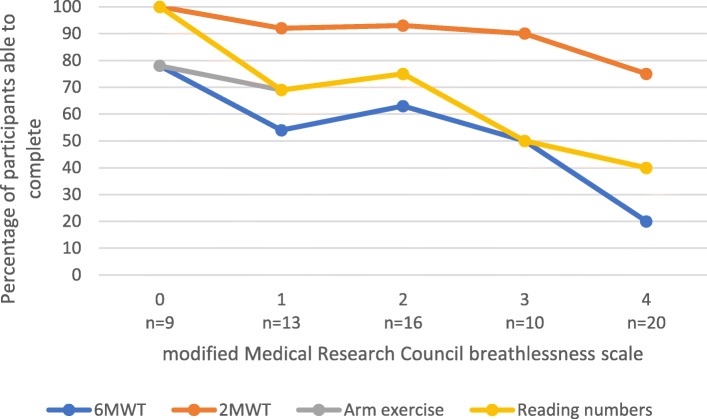
Percentage of completion of 6-min walk test, 2-min walk test, arm exercise and reading numbers by modified Medical Research Council breathlessness scale in 68 people with advanced cancer

As modified Medical Research Council breathlessness scores increased, the percentage of people able to complete the exercise tests declined (Fig. 2). For those people with an mMRC of 4, 20% were able to complete the 6MWT; 75% the 2MWT; and 40% arm exercises and reading numbers.

## Discussion

Three distinct levels of function defined people’s ability to participate in exercise tests that were expected to be limited by breathlessness: those who could complete walking tests (AKPS 70–90); those who could complete arm exercises (AKPS ≥50) and those who could only read numbers (AKPS ≥20). This work builds on the test devised by Wilcock et al. by defining populations for whom no other test is available. The functional cut off points for these exercise tests in advanced disease have not previously been defined.

This is the first study to try and determine which exercise test should be used to assess exertion-induced breathlessness as functional status declines. Measuring the level of, or changes in degree of exertion required to induce breathlessness is important in research about this symptom in people at the end of life. Percentage completion rates are an important first step in understanding the best test to use. Reproducibility of outcomes within a single level of function becomes the next important factor to consider and, finally, the ability to see change between groups as function declines.

Given the findings in this study, in the past, clinicians may have under-estimated the impact of an individual’s exertional limitations as function declines. Asking what people avoid in order to ensure they do not experience breathlessness is a clinically important question in this setting.

For future research, the findings of this current study suggest that if the population whose exertion limited by breathlessness is being studied includes a relatively large proportion of people with an AKPS of 20–40, then reading numbers would be the tool of choice; if above 40, then arm exercises would be appropriate. Walking tests are for only for the group of people who are functioning independently.

### Limitations

This was a relatively small convenience sample with understandably limited participation for people with poorer functional status. These data were only cross-sectional and a future longitudinal study would add value to the findings. One challenge of this clinician-initiated study was the time taken to recruit 68 participants and the even greater challenge of recruiting people with poor functional status.

### Strengths

This is a cohort that represents people with the full spectrum of function decline seen in the course of advancing cancer. This is the first such study and the findings confirm the importance of applying commonly used exercise tests (such as 6MWT) to a population who can reasonably participate in the measurement.

### Implications for clinical care

This study is primarily about reflecting which exercise tests should be used for assessing the exertion required to either induce breathlessness or limit function for clinical research in the future. The most direct clinical application of these findings is that the limitations imposed by declining functional status may well be under-estimated by clinicians. These are timely data stressing the level of function reflected in a measure such as the AKPS.

### Implications for research

Longitudinal data to assess these same parameters will allow intra-individual comparisons over time in order to assess responsiveness to change, and strengthen the understanding of the choices that should be made in assessing these patient populations.

## Conclusions

The results of this study demonstrate how functionally limited people are once their AKPS begins to fall. People become profoundly functionally impaired as disease progresses. When researching breathlessness in the population with advanced cancer, this study guides the choice of test to use at different points along their trajectory of decline through reporting the functional cut off points for four exercise-based tools.

This study demonstrates the importance of tailoring objective exercise-induced breathlessness tests to a person’s level of function in future breathlessness research in life-limiting illness.

## Abbreviations

2MWD: 2 min walk distance
6MWD: 6 min walk distance
2MWT: 2 min walk test
6MWT: 6 min walk test
AKPS: Australia-modified Karnofsky Performance Status
mMRC: modified Medical Research Council breathlessness scale
